# NFKBIZ regulates NFκB signaling pathway to mediate tumorigenesis and metastasis of hepatocellular carcinoma by direct interaction with TRIM16

**DOI:** 10.1007/s00018-024-05182-7

**Published:** 2024-04-06

**Authors:** Danfeng Guo, Ming Zhang, Tingju Wei, Xiaodan Zhang, Xiaoyi Shi, Hongwei Tang, Mingjie Ding, Jie Li, Shuijun Zhang, Wenzhi Guo

**Affiliations:** 1 Department of Hepatobiliary and Pancreatic Surgery, The First Affiliated Hospital of Zhengzhou University, Zhengzhou, 450052 Henan China; 2 Henan Key Laboratory for Digestive Organ Transplantation, The First Affiliated Hospital of Zhengzhou University, Zhengzhou, 450052 Henan China; 3 Department of Cardiac Surgery, The First Affiliated Hospital of Zhengzhou University, Zhengzhou, 450052 Henan China

**Keywords:** NFKBIZ, HCC, Ubiquitination, TRIM16, Sorafenib sensitivity

## Abstract

Hepatocellular carcinoma (HCC) is a malignant tumor with high incidence and mortality rates. NFKBIZ, a member of the nuclear factor kappa B inhibitory family, is closely related to tumor progression. However, the precise role of NFKBIZ in HCC remains unclear. To explore this, we conducted a series of experiments from clinic to cells. Western blot and qPCR revealed a significant downregulation of NFKBIZ in human HCC tissues. Clinical character analysis showed that the patients with lower NFKBIZ expression had poorer prognosis and higher clinical stage. By using CCK-8, wound healing, transwell invasion and migration assay, we discovered that NFKBIZ expression was reversely associated with the proliferation, invasion, and migration ability of HCC cells in vitro. Additionally, the results obtained from xenograft assay and lung metastasis models showed that NFKBIZ overexpression inhibited the growth and metastasis of HCC cells in vivo. Western blot and immunofluorescence assay further revealed that NFKBIZ mediated HCC cell growth and migration by regulating NFκB signaling transduction. Finally, flow cytometry, protein degradation assay and Co-immunoprecipitation indicated that TRIM16 can enhance NFKBIZ ubiquitination by direct interactions at its K48 site, which may thereby alleviate HCC cell apoptosis to induce the insensitivity to sorafenib. In conclusion, our study demonstrated that NFKBIZ regulated HCC tumorigenesis and metastasis by mediating NFκB signal transduction and TRIM16/NFKBIZ/NFκB axis may be the underlying mechanism of sorafenib insensitivity in HCC.

**Supplementary Information:**

The online version contains supplementary material available at 10.1007/s00018-024-05182-7.

## Introduction

Hepatocellular carcinoma (HCC) is the most prevalent primary liver cancer, accounting for approximately 70–80% of liver cancer cases. It ranks sixth in cancer incidence and third as the leading cause of cancer-related deaths [1]. Current treatment options for HCC include surgical resection, liver transplantation, radiotherapy, and chemotherapy. However, these therapeutic methods still face challenges, such as individual variability, chemo- and radio-resistance, leading to limited overall benefits [2–4]. Therefore, exploring new and improving present treatment methods for HCC are of great significance.

NFKBIZ belongs to the nuclear factor kappa B inhibitory (IκB) family and exerts an important effect on various biological activities including individual immunity and inflammatory response [5]. Generally, the IκB proteins inhibit the nuclear translocation of cytoplasmic NFκB, whereas NFKBIZ inhibits activated NFκB signaling by binding to NFκB in the cell nucleus, ultimately resulting in the inactivation of NFκB downstream genes [6]. NFKBIZ played a bidirectional role in the progression of divergent tumors. Research on diffuse large B-cell lymphoblastoma (DLBCL) proposed that approximately 40% of primary testicular DLBCL (PT-DLBCL) patients were accompanied by NFKBIZ somatic cell mutations, and the abnormal expression of NFKBIZ promoted PT-DLBCL advancement [7, 8]. Notably, the mutation rate of NFKBIZ was higher in intestinal epithelial cells in the context of ulcerative colitis, but lower in colitis-led tumor cells, suggesting that intestinal epithelial cells may resist tumor transformation through selective NFKBIZ mutation [9]. Studies on rectal cancer showed that NFKBIZ mutations were associated with rectal cancer radiation resistance [10]. Relative research of bladder cancer showed NFKBIZ exerted an antitumoral effect by modulating PTEN/PI3K/Akt signaling pathway [11]. Previous researches have also indicated the intimate correlation between dysregulated expression of NFKBIZ and liver diseases such as acute hepatitis, nonalcoholic fatty liver disease and HBV- related HCC [12, 13]. Mechanistically, NFKBIZ downregulation can suppress the expression of IL-6 (a hepatoprotective cytokine) and resulted in restricted inhibition of hepatitis; NFKBIZ can also regulate triglyceride metabolism to attenuate the progression of nonalcoholic fatty liver disease. In addition, NFKBIZ was reported to inhibit the progression of HBV-related HCC, whereas further investigation was required for the detailed mechanism underlying the progression of HCC.

Sorafenib is a multi-target tyrosine kinase inhibitor currently used as a first-line treatment for the advanced HCC. Sorafenib is recognized to be beneficial for the overall survival (OS) of HCC patients, but there are limitations remaining unresolved. Nowadays, drug resistance and insensitivity of sorafenib were the major problems increasingly emerged, leading to the unsatisfactory therapeutic effect on HCC patients [14]. Mechanistically, sorafenib inhibited the signaling transduction of the Raf/MAPK/ERK pathway by binding to receptor tyrosine kinases [15]. The development of sorafenib resistance is involved in the activation of bypass pathways that intermediately activated the downstream targets of MAPK signaling pathway [16]. Studies have indicated that the p38-MAPK, PI3K/AKT, and NFκB signaling pathways were aberrantly activated when HCC cells gained sorafenib resistance [17]. Since the mechanism of sorafenib insensitivity was complex and not fully understood, it was of great significance to identify key targets to solve the problem for HCC patients.

Here, we aimed to investigate the impact of NFKBIZ on HCC and clarify the relative mechanism. Our research revealed that low NFKBIZ level indicated a poor prognosis for HCC patients and NFKBIZ modulated the proliferation and metastasis of HCC cells via regulating NFκB pathway. We discovered TRIM16 alleviated the inhibitory effect of NFKBIZ on HCC cells by increasing NFKBIZ ubiquitination. NFKBIZ overexpression enhanced the apoptosis rate of HCC cells, which can be mitigated by TRIM16 through promoting NFKBIZ degradation under sorafenib treatment. These findings suggested the potential role of NFKBIZ as a new target for HCC treatment and prognosis as well as the key target to guard the sensitivity of HCC to sorafenib.

## Materials and methods

### NFKBIZ expression profile mining and bioinformatics analysis in The Cancer Genome Atlas (TCGA)

Relevant data or figures from TCGA database (http://cancergenome.nih.gov) were downloaded and analyzed using the Gene Expression Profiling Interacting Analysis (GEPIA; http://gepia.cancer-pku.cn) and UALCAN (http://ualcan.path.uab.edu) database.

### Human HCC tissue specimens and cell lines

A total of 102 pairs of human liver cancer and paired noncancerous tissues were randomly obtained from HCC patients who received surgical treatment in the First Affiliated Hospital of Zhengzhou University, China. All participants did not undergo any radiotherapy or chemotherapy before surgical resection. Patient information was collected and followed up throughout the entire time span from 2012 to the present. The domestic information and clinical characteristics of HCC patients were demonstrated in Table 1. Human HCC cell lines including MHCC97H, HepG2 and Huh7 were obtained from Henan Organ Transplantation Research Center.

**Table 1:** The correlation between NFKBIZ and the clinical characters of HCC patients

Clinicopathological characteristics	Total (*n* = 102)	NFKBIZ expression		*p* value
		Negative	Positive	
Age				
≥55	45	21	24	0.55
<55	57	30	27	
Sex				
Female	16	8	8	1
Male	86	43	43	
Serum AFP				
≤20 ng/ml	40	22	18	0.417
>20 ng/ml	62	29	33	
HBV				
Absent	16	4	12	0.029*
Present	86	47	39	
Cirrhosis				
Absent	26	16	10	0.173
Present	76	35	41	
Tumor number				
Single	74	35	39	0.375
Multiple	28	16	12	
Maximal tumor size				
≤5 cm	66	27	39	0.013*
>5 cm	36	24	12	
Tumor differentiation				
I–II	77	38	39	0.818
III–IV	25	13	12	
Microvascular invasion				
Absent	68	33	35	0.674
Present	34	18	16	
Tumor encapsulation				
Absent	85	42	43	0.79
Present	17	9	8	
Extrahepatic metastasis				
Absent	90	44	46	0.539
Present	12	7	5	
Portal vein thrombus				
Absent	84	43	48	0.11
Present	18	8	3	
Recurrence				
Absent	62	26	36	0.043*
Present	40	25	15	

The bold values reflected that the clinical pathological characteristics had statistical significance between the NFKBIZ-Negative and NFKBIZ-Positive group

*p < 0.05

### Quantitative real-time PCR assay (qRT-PCR)

The total RNA of HCC cells and clinical tissue samples were extracted by Trizol, and HiFiScript^®^ III First Strand cDNA Synthetic Kit was used to reverse transcribed mRNA into cDNA. The 2× SYBR Green qPCR Master Mix and Rox were used for qRT-PCR. The mRNA expression level was calculated using the standard formula, 2^−ΔΔCT^ = (CT_target_ − CT_β-actin_) experiment- (CT_target_ − CT_β-actin_) control, to evaluate the fold change. All primer sequences used were summarized in Table 2.

**Table 2:** Information for the reagent and resource

Reagent and resource	Source	Identifier
*Antibodies*		
NFKBIZ (1:1000)	Proteintech, China	14,014–1-AP
GAPDH (1:1000)	Proteintech, China	10,494–1-AP
TRIM16 (1:1000)	Proteintech, China	24,403–1-AP
Anti-Flag (1:1000)	Proteintech, China	66,008–4-Ig
Anti-HA (1:1000)	MBL, Japan	M180-3MS
Anti-Myc (1:1000)	MBL, Japan	M047-8
E-Cadherin (1:1000)	Wanleibio, China	WL01482
N-Cadherin (1:1000)	Wanleibio, China	WL01047
Snail (1:1000)	Wanleibio, China	WL01863
Survivin (1:1000)	Proteintech, China	10,508–1-AP
Vimentin (1:1000)	Proteintech, China	10,366–1-AP
BCL-2 (1:1000)	Proteintech, China	68,103–1-Ig
Caspase3/c-caspase3 (1:1000)	Proteintech, China	19,677–1-AP
Bax (1:1000)	Proteintech, China	50,599–2-Ig
IKKα/β (1:1000)	Abcam, UK	ab194528
p65 (1:1000)	Proteintech, China	80,979–1-RR
p-p65 (1:1000)	Proteintech, China	82,335–1-RR
IKBα (1:1000)	Proteintech, China	10,268–1-AP
p-IKBα (1:1000)	Proteintech, China	82,349–1-RR
MMP9 (1:1000)	Wanleibio, China	WL03096
CyclinD1 (1:1000)	Wanleibio, China	WL01435a
C-Myc (1:1000)	Wanleibio, China	WL01781
PCNA (1:1000)	Proteintech, China	10,205–2-AP
β-Catenin (1:1000)	Proteintech, China	51,067–2-AP
p-AKT (1:1000)	Wanleibio, China	WLP001a
AKT (1:1000)	Wanleibio, China	WL0003b
Goat Anti-Rabbit IgG (H + L) Alexa Fluor 594 (1:500)	Abways, China	AB0151
HRP-conjugated Affinipure Goat Anti-Mouse IgG (H + L) (1:10,000)	Proteintech, China	SA00001-1
HRP-conjugated Affinipure Goat Anti-Rabbit IgG (H + L) (1:10,000)	Proteintech, China	SA00001-2
*Experiment models: cell lines*		
BALB/C nude mice	Beijing Vital River Laboratory Animal Technology (China)	N/A
HepG2	Danfeng Guo	N/A
MHCC97H	Danfeng Guo	N/A
Huh7	Danfeng Guo	N/A
*Oligonicleotides and plasmids*		
NFKBIZ F: ACACCCACAAACCAACTCTGG	SUNYA, China	N/A
NFKBIZ R: GGCAAAACTGTGATTCTGGACC	SUNYA, China	N/A
TRIM16 F: GTCCTGTCTAACCTGCATGGT	SUNYA, China	N/A
TRIM16 R: GGCAGTATCGCCAGTTGTG	SUNYA, China	N/A
TRIM28 F: TTTCATGCGTGATAGTGGCAG	SUNYA, China	N/A
TRIM28 R: GCCTCTACACAGGTCTCACAC	SUNYA, China	N/A
TRIM31 F: AACCTGTCACCATCGACTGTG	SUNYA, China	N/A
TRIM31 R: TGATTGCGTTCTTCCTTACGG	SUNYA, China	N/A
TRIM43 F: AGGGAACCATCACCGAAAATG	SUNYA, China	N/A
TRIM43 R: TTGTTTGCCTATGGGTCCCAC	SUNYA, China	N/A
TRIM63 F: CTTCCAGGCTGCAAATCCCTA	SUNYA, China	N/A
TRIM63 R: ACACTCCGTGACGATCCATGA	SUNYA, China	N/A
TRIM9 F: GTGTGCGGCTCCTTCTATCGA	SUNYA, China	N/A
TRIM9 R: GCTGTATAGGCTCATCTTGTCCA	SUNYA, China	N/A
GAPDH F: GTGGATATTGTTGCCATCAA	SUNYA, China	N/A
GAPDH R: ATTCGTTGTCATACCAGGAA	SUNYA, China	N/A
*sh-NFKBIZ (sh-1)*		
5′-AGAAAGCAAATTGGCAAATATTTCAAGAGAATATTTGCCAATTTGCTTTCTTT-3′	GenePharma, China	–
*sh-NFKBIZ (sh-2)*		
5′-CACTTCACATGCTGGATATTATTCAAGAGATAATATCCAGCATGTGAAGTGTT-3′	GenePharma, China	–
*sh-Negative Control (shNC)*		
5′-TTCTCCGAACGTGTCACGTTTCAAGAGAACGTGACACGTTCGGAGAATT-3′	GenePharma, China	–
pGPU6/GFP/Neo-homo-GAPDH (shNC)	GenePharma, China	–
pSLenti-EF1-EGFP-P2A-Puro-CMV-NFKBIZ-3xFLAG-WPRE (NFKBIZ)	OBiO Technology, China	–
pSLenti-EF1-EGFP-P2A-Puro-CMV-MCS-3xFLAG-WPRE (Vector)	OBiO Technology, China	–
pSLenti-EF1-EGFP-P2A-Puro-CMV-TRIM16-HA-WPRE (TRIM16)	GenePharma, China	–
pSLenti-EF1-EGFP-P2A-Puro-CMV-MCS-HA-WPRE (Vector)	GenePharma, China	–
pSLenti-EF1-EGFP-P2A-Puro-CMV-Ubi-K48O-Myc-WPRE (Myc-Ubi-K48O)	GenePharma, China	–
pSLenti-EF1-EGFP-P2A-Puro-CMV-Ubi-K48R-Myc-WPRE (Myc-Ubi-K48R)	GenePharma, China	–
pSLenti-EF1-EGFP-P2A-Puro-CMV-Ubi-WT-Myc-WPRE (Myc-Ubi-WT)	GenePharma, China	–
pCMV-dR8.91	GenePharma, China	–
pCMV-VSV-G	GenePharma, China	–
*Reagents*		
Phorbol 12-myristate 13-acetate (PMA, concentration:50ng/ml)	MedChemExpress, US	–
Cycloheximide (CHX, concentration:50ng/ml)	MedChemExpress, US	–
MG132 (concentration:50ng/ml)	MedChemExpress, US	–
Chloroquine (CQ, concentration:50ng/ml)	MedChemExpress, US	–
Lipo2000	Biosharp, China	–
Cell Counting Kit-8 (CCK8)	Solarbio, China	–
PMSF	Solarbio, China	–
Protein Inhibitor Cocktail	Thermo Fisher Scientific, US	–
BCA Protein Assay reagent	Solarbio, China	–
Trizol	Invitrogen, US	–
Annexin V-APC/7-AAD Apoptosis Detection Kit	KeyGEN BioTECH, China	–
HiFiScript^®^ III First Strand cDNA Synthetic Kit	Vazyme, China	–
2× SYBR Green qPCR Master Mix	Bimake, US	–

### Western blot assay

The tissue and cell proteins were lysed in RIPA buffer supplemented with 1mM PMSF and Protein inhibitor Cocktail, and in turn samples were put on the ice for 30 min. The protein concentration was measured by using BCA Protein Assay reagent. An equal amount of protein (10 µg/lane) was separated by 10% SDS-PAGE and subsequently transferred to the PVDF membrane and then sealed in 5% skimmed milk powder for 60 min. The membrane was incubated overnight with a specific antibody at 4℃. The next day, the membrane was incubated with the corresponding anti-HRP antibody at room temperature for 2 h. Image J software was utilized for densitometric analysis. All the antibodies used were presented in Table 2.

### Chemicals, transient transfection, and lentivirus infection

MHCC97H, Huh7, and HepG2 cells (4 × 10^5^ per well) were planted in 6-well plates, and then added with the mixtures of plasmid and transfection reagent. Samples were collected after 48 h. The plasmids, pCMV-dR8.91 and pCMV-VSV-G, were utilized for lentivirus packaging in order to achieve stable NFKBIZ overexpression. 293T cells (3 × 10^5^ cells per well) were seeded in 6-well plates to reach 90% confluence the following day. After co-transfecting pCMV-dR8.91, pCMV-VSV-G, and the targeted plasmids (2μg per plasmid) for 48 h, the supernatant containing sufficient lentivirus was collected and used to infect MHCC97H, Huh7, and HepG2 cells in 6-well plates (MOI 40). After 24 h, the infected cells were selected and further cultured in medium containing 1μg/mL puromycin. All the drug were presented in Table 2 with specific information including the concentration of use. All experiments were performed with mycoplasma-free cells. All human cell lines have been authenticated using STR (or SNP) profiling within the last 3 years.

### Cell proliferation and apoptosis assay

Transient transfected MHCC97H, Huh7, and HepG2 cells (1 × 10^3^ cells per well) were developed in 96 well plates and observed at 0, 24, 48, 72, and 96 h, respectively. Cell viability was evaluated by CCK-8 analysis. At the designated time point, the culture medium was replaced with the fresh one containing 10% CCK-8. Following an incubation period at 37°C for 120 min, the absorbance of the samples was measured at 450nm. Flow cytometry and Annexin V-APC/7-AAD Apoptosis Detection Kit were used to evaluate the apoptotic level of HCC cells.

### Colony formation assay

The transfected MHCC97H, Huh7, and HepG2 cells (1 × 10^3^ cells per well) were implanted in the 6-well plates and cultured for 14 days. During the cultivation period, the intermediary was changed every 4 days. At the planned time point, colonies were rinsed with PBS, fixed with 4% paraformaldehyde for 20 min, and subsequently stained with 0.1% crystal violet for 30 min. and then cleaned with PBS and photographed.

### Wound healing assay

The transfected MHCC97H, Huh7, and HepG2 cells (4 × 10^6^ cells per well) were implanted in 6-well plates and cultured to about 90% confluence overnight. A 200μl sterile pipette tip was used to scratch the monolayer cells and afterwards cells were cultured in serum-free fresh medium. The width of the wound was captured and recorded at 0h, 24h, and 48h, respectively. The area of cell migration was measured using ImageJ software (v1.8.0).

### Cell migration and invasion assay

Transfected MHCC97H, Huh7, and HepG2 cells (2 × 10^5^ per well) were implanted in the upper chamber of Incorporated Cell Culture Insertions with a polycarbonate filtration film (8 μM aperture size, 6.5 mm diameter). In the migration experiment, 2 × 10^5^ cells were incubated in 200μl fresh FBS-free medium in the upper chamber without Matrigel, whereas with Matrigel for invasion experiment. 600μl DMEM containing 20% FBS was added in the lower chamber. After 2 days, the non-migrated and non-invaded cells located in the upper chamber were carefully wiped, while the migrated and invaded cells located in the lower chamber were treated with 4% paraformaldehyde and then stained with 0.1% crystal violet.

### Immunoprecipitation

Transfected MHCC97H, Huh7, and HepG2 cells were lysed by IP lysis buffer on ice. After 48 h, the pyrolysis product was centrifuged at 12,000 rpm for 10 min. A portion of the retained supernatant was taken for Input, and the rest portion was used for IP. After that, IP from each group was mixed with the primary antibody. The protein A and G agarose beads were cleaned three times with IP lysis buffer, then 30μl beads were added to the mixture of each group. The mixture was afterwards incubated overnight by a shaker at 4°C. Eventually, the beads of each group were collected and cleaned with IP lysis buffer, then added with 2× loading buffer and heated at 100℃ for 10 min.

### Immunohistochemical and immunofluorescence assay

Immunohistochemical (IHC) assay was conducted on paraformaldehyde-fixed paraffin sections. The detailed procedure and statistical evaluation were performed as previously described [16]. For immunofluorescence, MHCC97H and Huh7 cells (2 × 10^4^ cells per group) transfected with NFKBIZ overexpressed or control plasmids were implanted on the cover glass slides, respectively. After 48 h, the cells were washed twice with PBS, and then fixed with 4% paraformaldehyde for 20 min. After that, the cells were sealed into PBS with 0.5% Triton X-100 at room temperature for 20 min, and then added with 5% BSA. Afterwards, cells were incubated with p65 antibody (1:1000, 80,979–1-RR, Proteintech, China) and the specific second antibody. Cells were added with 4,6-diamino-2-phenylindole (DAPI) for 5 min and captured with fluorescence microscope.

### Xenograft assay and lung metastasis models

For xenograft assay, MHCC97H or Huh7 cells that were stably transfected with lentivirus vector or lentivirus vector overexpressing NFKBIZ were resuspended in 100 μl PBS, respectively, and subcutaneously injected into the back of 4-week-old male nude mice. To be specific, the mice from each group were injected with HCC cells stably expressing Vector on the left dorsal side, and those stably overexpressing NFKBIZ on the right dorsal side. For lung metastasis models, 5 × 10^6^ HCC cells in 200μl PBS was injected into the tail vein. 8 weeks later, mice were sacrificed to obtain the lung tissues for the counting of lung metastasis nodules and H&E staining. All mice were randomly grouped using random number method.

### Statistical analysis

All experiments in cells were conducted with three independent repetitions. All statistical results were processed using GraphPad Prism 8.0. The data from three independent repeated experiments were presented in a mean ± SD manner. The statistical methods used in each experiment were given in the figure legends. The relationship between NFKBIZ expression and various clinicopathological parameters was evaluated with the Mann–Whitney U-tests. The diversity was thought to have significance at * *p* < 0.05, ** *p* < 0.01, *** *p* < 0.001, **** *p* < 0.0001 compared to specific control group.

## Results

### NFKBIZ expression is associated with the prognosis of HCC patients

**Fig. 1:**
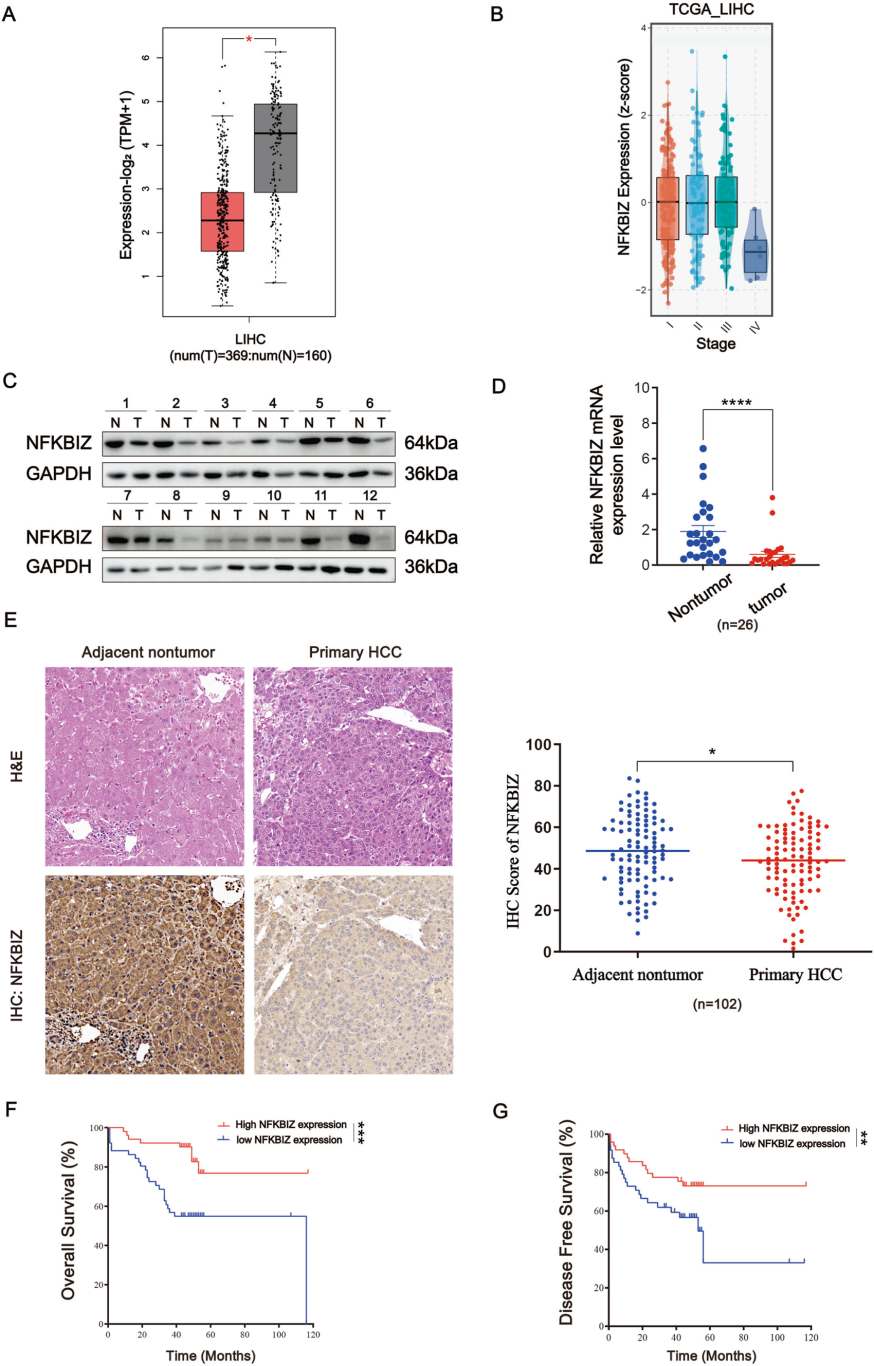
NFKBIZ is downregulated in HCC tissues and associated with the prognosis of patients. **A** NFKBIZ expression in LIHC based on sample types in the Gene Expression Profiling Interacting Analysis database (GEPIA) (T: tumor, *n* = 369; N: normal liver, *n* = 160, *p* < 0.05, Student’s *t* test). **B** The gene expression of NFKBIZ in LIHC at different HCC stages was presented according to TCGA (*p* = 0.0382, Kruskal–Wallis test). **C**,**D** Western blot (*n* = 12) and qRT-PCR (*n* = 26) were employed to detect the expression of NFKBIZ protein and mRNA in adjacent nontumor (N) and HCC tissue (T) (*p* < 0.0001, Wilcoxon matched-pairs signed rank test). **E** Immunohistochemical assay was used to compare the expression of NFKBIZ in adjacent nontumor tissue with that in the primary HCC tissue from our cohort, corresponding H&E images were also presented (*n* = 102, magnification: 100×, *p* = 0.0274, Student’s *t* test). **F**,**G** HCC patients were classified into low- and high-NFKBIZ group (*n* = 51, respectively) by the cut point, 50%, according to individual IHC score. Kaplan–Meier survival analysis was used to evaluate the effect of NFKBIZ on the OS (*n* = 102, *p* = 0.0007, Kaplan–Meier survival analysis) and DFS (*n* = 102, *p* = 0.0345, Kaplan–Meier survival analysis) between the two groups. * *p* < 0.05, ** *p* < 0.01, *** *p* < 0.001, **** *p* < 0.0001 compared to specific control group

We started with the TCGA database to predict NFKBIZ mRNA levels in HCC samples and observed a significant downregulation of NFKBIZ compared to normal liver tissues (Fig. 1A). Additionally, NFKBIZ was found to be negatively correlated with HCC stage (Fig. 1B). To validate the observation from the TCGA database, we performed Western blot and qRT-PCR to detect NFKBIZ expression in HCC and paired adjacent noncancerous tissues in our cohort. The results showed a consistent downregulation of both NFKBIZ protein and mRNA levels in HCC tissues (Fig. 1C, D). Next, we conducted the IHC assay on HCC tissue specimens from 102 patients. The results revealed reduced NFKBIZ expression in HCC tissues compared to adjacent nontumoral tissues (Fig. 1E). To clarify the clinical significance of NFKBIZ in HCC, patients were divided into NFKBIZ high- and low-expression groups by H-SCORE at the cutoff point of 50%. Prognostic statistics showed that HCC patients with high expression of NFKBIZ possessed remarkably prolonged overall survival (OS) and disease-free survival (DFS) (Fig. 1F, G). Clinical character analysis indicated that NFKBIZ high expression was significantly related to reduced HCC recurrence rate, lower HBV infection rate and decreased tumor size (Table 1). In summary, our data revealed the downregulation of NFKBIZ in HCC cells and suggested NFKBIZ had the potential correlation with HCC progression.

### NFKBIZ affects proliferation, metastasis and EMT in HCC cells

**Fig. 2:**
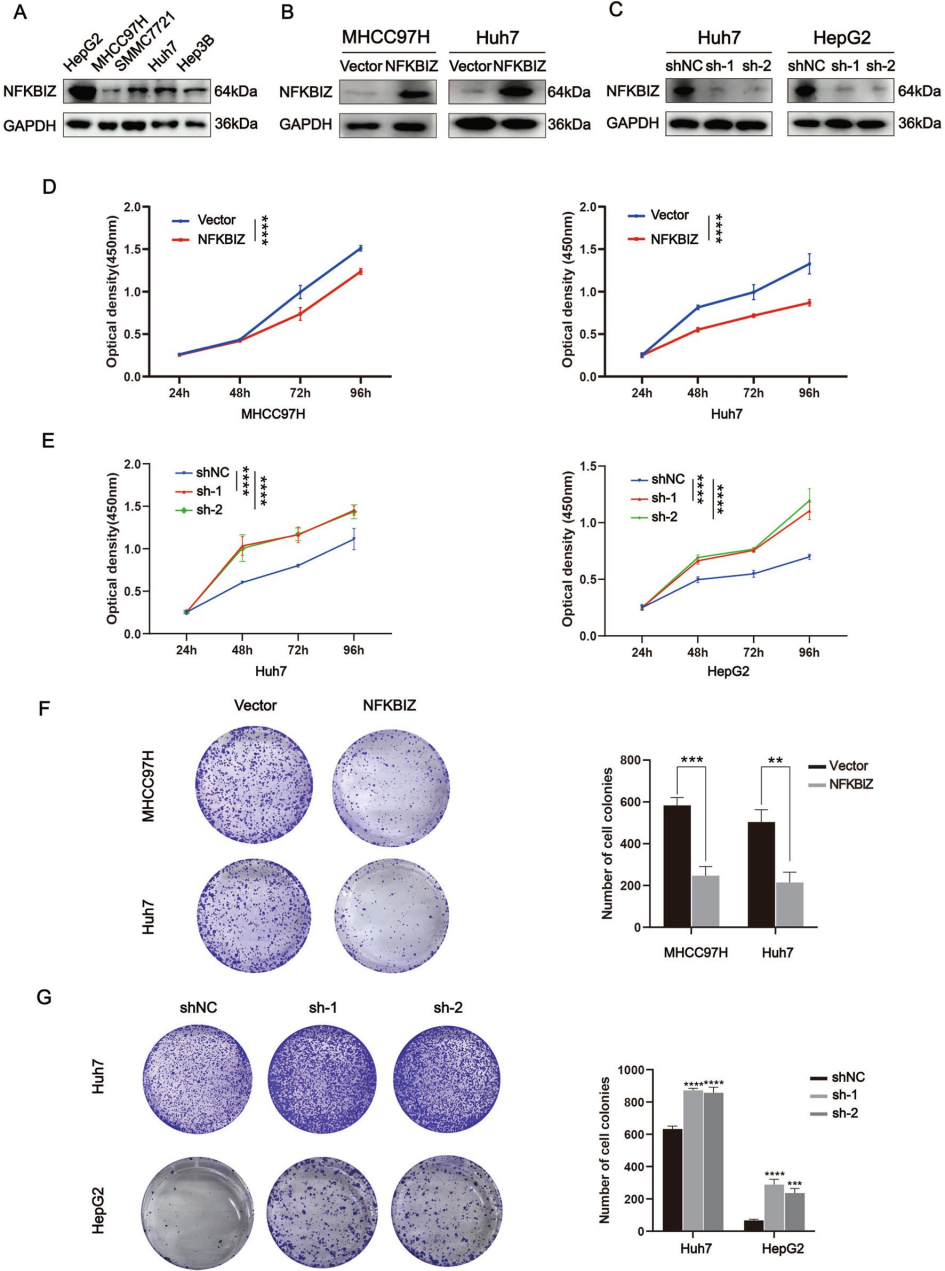
NFKBIZ regulates the proliferative ability of HCC cells in vitro. **A** NFKBIZ expression in HCC cell lines (HepG2, MHCC97H, SMMC7721, Huh7, Hep3B) was examined (*n* = 3 for each group). **B** The expression of NFKBIZ in MHCC97H and Huh7 cells was checked after the transfection with Vector or NFKBIZ overexpression plasmid for 24h (*n* = 3 for each group). **C** The expression of NFKBIZ in Huh7 and HepG2 cells with transient transfections of shNC, sh-NFKBIZ-1, and sh-NFKBIZ-2 plasmid was checked at 48 h (*n* = 3 for each group). **D**,**E** Cellular proliferative ability was examined via the CCK-8 analysis, showing as the optical density in MHCC97H, Huh7 and HepG2 cells with different treatments mentioned above at the time points of 24h, 48h, 72h and 96h (*n* = 5 for each group, **D** (left): *p* < 0.0001, **D** (right): *p* < 0.0001; **E** (left, sh-NC vs. sh-1): *p* < 0.0001, **E** (left, sh-NC vs. sh-2): *p* < 0.0001; **E** (right, sh-NC vs. sh-1): *p* < 0.0001, **E** (right, sh-NC vs. sh-2): *p* < 0.0001, one-way ANOVA). **F**,**G** Representative images exhibiting the colony formed in MHCC97H, Huh7 and HepG2 cells with different treatments mentioned above, which were photographed by optical microscope. Histograms showing the number of cell colonies in these cell lines were also presented (**F** (MHCC97H), *p* = 0.0005, **F** (Huh7), *p* = 0.0029, Student’s *t* test; **G** (sh-NC vs. sh-1, Huh7), *p* < 0.0001, **G** (sh-NC vs. sh-2, Huh7), *p* < 0.0001; **G** (sh-NC vs. sh-1, HepG2), *p* < 0.0001, **G** (sh-NC vs. sh-2, HepG2), *p* = 0.0003, one-way ANOVA). ** *p* < 0.01, *** *p* < 0.001, **** *p* < 0.0001 compared to specific control group

To investigate the impact of NFKBIZ on HCC progression, we then assessed the expression of NFKBIZ in HCC cell lines. Among the five cell lines, NFKBIZ showed the lowest expression in MHCC97H cells, the highest expression in HepG2 cells, and a relatively average expression in Huh7 cells (Fig. 2A). Accordingly, we performed NFKBIZ overexpression in MHCC97H and Huh7 cells, and conducted NFKBIZ silencing in Huh7 and HepG2 cells. Figure 2B and C showed the successful establishment of stable NFKBIZ overexpression in MHCC97H and Huh7 cell lines, as well as transient NFKBIZ silencing in Huh7 and HepG2 cells. Subsequently, we evaluated the effect of NFKBIZ on HCC proliferation. There was a delayed rate of cell growth and colony formation in the HCC cells with NFKBIZ overexpression (Fig. 2D, F), indicating the proliferation was inhibited. In contrast, NFKBIZ silencing significantly enhanced the proliferation of HCC cells, showing as the larger colonies forming at an accelerated rate (Fig. 2E, G).

**Fig. 3:**
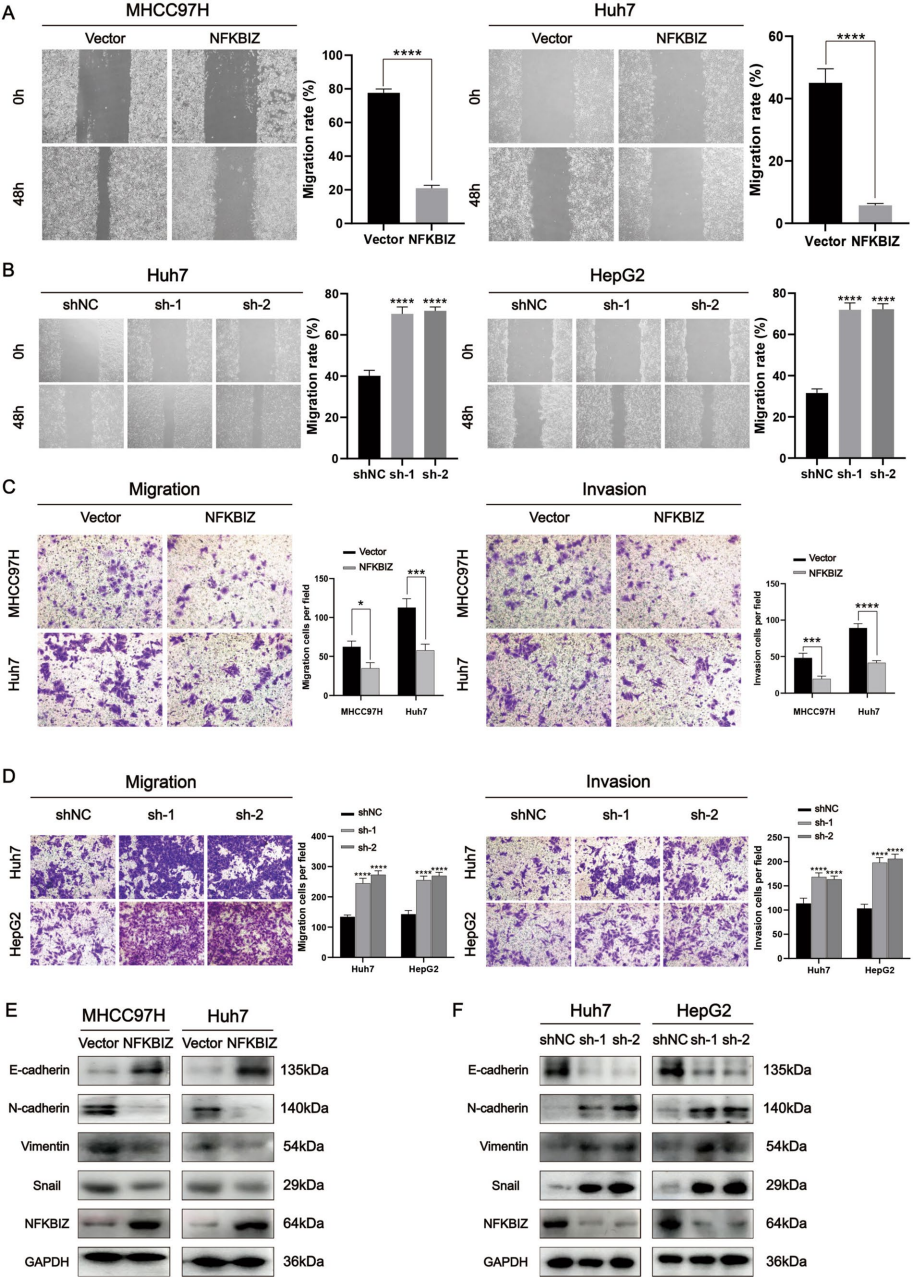
NFKBIZ modulates the migration and invasion of HCC cells through affecting EMT in vitro. **A**,**B** The migratory activity of HCC cells with NFKBIZ overexpression or silencing was examined by wound healing assay. The wound space in different groups of HCC cells was photographed at 0h and 48h by optical microscope (magnification: 40×, *n *= 3 for each group). **A** (left, *p* < 0.0001, Student’s *t* test); **A** (right, *p* < 0.0001, Student’s *t* test); **B** (left, sh-NC vs. sh-1, Huh7, *p* < 0.0001); **B** (left, sh-NC vs. sh-2, Huh7, *p* < 0.0001); **B** (right sh-NC vs. sh-1, HepG2, *p* < 0.0001); **B** (right, sh-NC vs. sh-2, HepG2, *p* < 0.0001, one-way ANOVA). **C**,**D** Transwell assays showed the migratory and invasive capacity of HCC cells with NFKBIZ overexpression or knockdown by optical microscope (magnification: 40×, *n* = 3 for each group, **C** (left, MHCC97H, *p* = 0.0205; Huh7, *p* = 0.0003, Student’s *t* test); **C** (right, MHCC97H, *p* = 0.0007; Huh7, *p* < 0.0001, Student’s *t* test); **D** (left, Huh7, sh-NC vs. sh-1, *p* < 0.0001; sh-NC vs. sh-2, *p* < 0.0001); **D** (left, HepG2, sh-NC vs. sh-1, *p* < 0.0001; sh-NC vs. sh-2, *p* < 0.0001, one-way ANOVA); **D** (right, Huh7, sh-NC vs. sh-1, *p* < 0.0001; sh-NC vs. sh-2, *p* < 0.0001; right, HepG2, sh-NC vs. sh-1, *p* < 0.0001; sh-NC vs. sh-2, *p* < 0.0001, one-way ANOVA). **E**,**F** The EMT-associated proteins, E-cadherin, N-cadherin, Vimentin and Snail were analyzed via Western blot 48 h after overexpressing or knocking down NFKBIZ in MHCC97H, Huh7 and HepG2 cells. * *p* < 0.05, *** *p* < 0.001, **** *p* < 0.0001 compared to specific control group

It’s well-known that a poorer HCC prognosis is largely associated with a higher invasive and metastatic capacity. We further inquiry whether NFKBIZ affected HCC migration and invasion, although there was no statistical significance in the correlation between NFKBIZ expression and extrahepatic metastasis in our clinical character analysis. We conducted the wound healing test to examine the impact of NFKBIZ on the migratory ability of HCC cells. A slower enhancement of the wound healing area was observed in HCC cells overexpressing NFKBIZ compared to the Vector group (Fig. 3A), which was consistent with the finding from the transwell migration experiment (Fig. 3C). Conversely, the migration rate of HCC cells with NFKBIZ silenced was notably higher compared to those of shNC group (Fig. 3B), in line with the transwell migration experiment results (Fig. 3D). Besides, the transwell invasion assay demonstrated that NFKBIZ overexpression inhibited the invasion ability of HCC cells, while NFKBIZ silencing had the opposite effect (Fig. 3C, D).

To explore the underlying mechanisms that NFKBIZ affected HCC migration and invasion, we investigated the correlation between NFKBIZ and epithelial-mesenchymal transition (EMT), which is closely linked to tumor metastasis and invasion [18]. Western blot revealed a negative correlation between NFKBIZ and the EMT-related proteins. Specifically, in HCC cells with high expression of NFKBIZ, there was a reduction of mesenchymal markers such as N-cadherin, Vimentin, and Snail (Fig. 3E). While the expression level of epithelial cell marker, E-cadherin, was decreased under the condition of NFKBIZ silencing (Fig. 3F). Therefore, these data suggested that NFKBIZ may regulate the metastatic ability of HCC by altering EMT.

### NFKBIZ inhibits the growth and metastasis of HCC cells in vivo

**Fig. 4:**
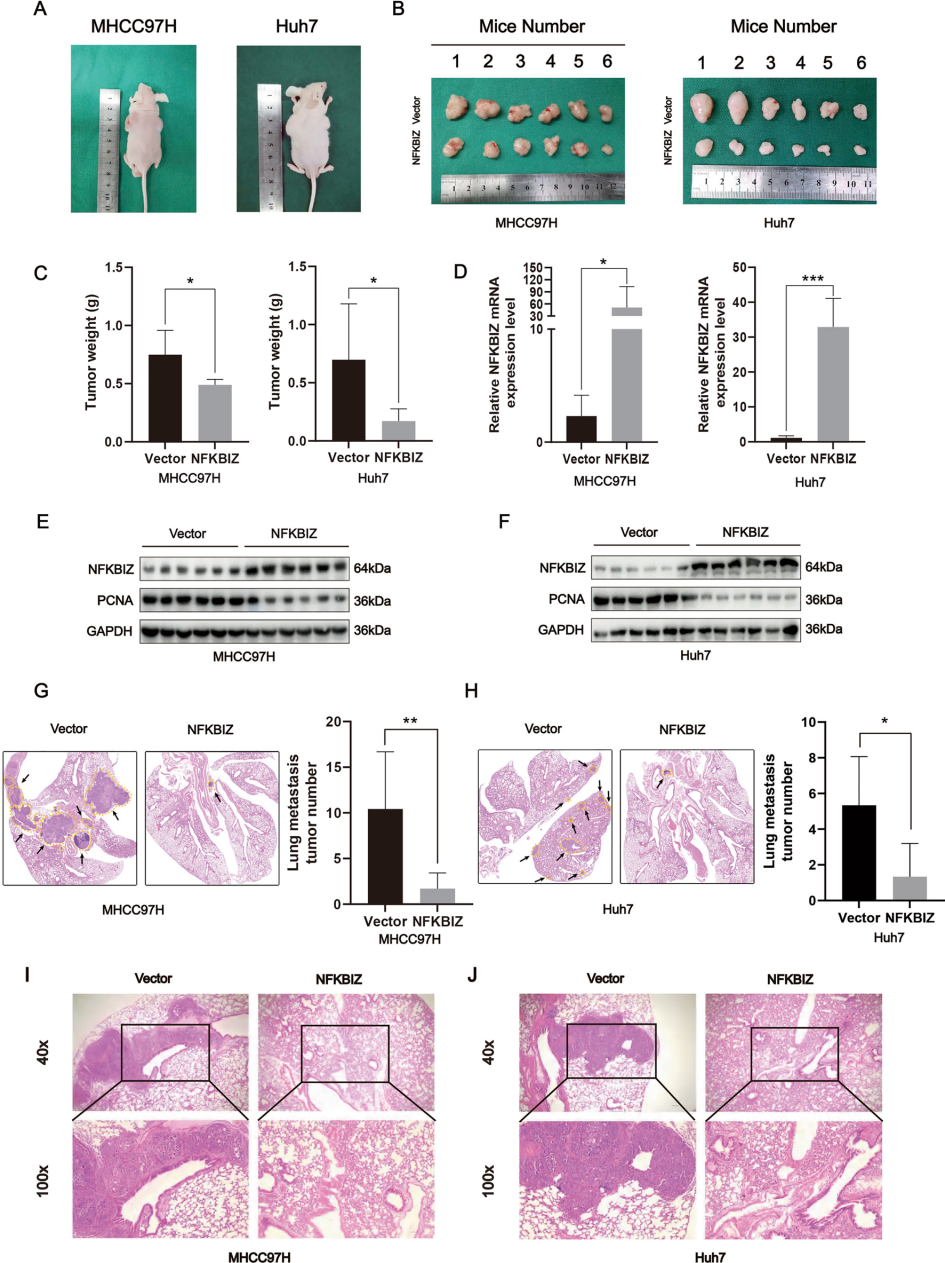
NFKBIZ inhibits HCC cell growth and metastasis in vivo. Representative images of BALB/c-nude mice (**A**) in each group (*n* = 6) and the tumor nodules (**B**) at 21 days after subcutaneous injections of MHCC97H or Huh7 cells (5 × 10^6^ cells resuspended in 100μl PBS) with Vector on the left (Vector group) or NFKBIZ overexpression on the right (NFKBIZ group). **C** The weight of tumors in each group of mice in **A.** Left, MHCC97H, *p* = 0.0270; Right, Huh7, *p* = 0.0427, Welch’s *t* test). **D** The mRNA level of NFKBIZ in tumor tissues extracted from subcutaneous mouse xenograft models in each group of mice in **A.** Left, MHCC97H, *p* = 0.0494; Right, Huh7, *p* = 0.0002, Welch’s *t* test). **E**,**F** NFKBIZ and PCNA expressions in the tumor tissue from subcutaneous mouse xenograft models in each group of mice in **A**. **G**,**H** Lung metastasis models were established by the tail vein injection of 5 × 10^6^ MHCC97H or Huh7 cells resuspended in 200μl PBS for 8 weeks. **G** Representative H&E images of lung tissues with tumor metastasis (magnification: 10×). **H** Statistical analysis of metastatic nodules from the lung tissues was also presented (*n* = 6 for each group, **G** *p* = 0.0096; **H** *p* = 0.0162, Welch’s *t* test). **I**,**J** Representative H&E images of lung tissues from those in **G** and **H**, photographed by optical microscope (Magnification: 40× above, 100× below). * *p* < 0.05, ** *p* < 0.01, *** *p* < 0.001 compared to specific control group

To further verify the effect of NFKBIZ on tumor formation in vivo, we constructed the xenograft mouse model by subcutaneously injecting MHCC97H and Huh7 with stable Vector expression and NFKBIZ overexpression into the left and right back of nude mice, respectively. Compared to the tumor tissue from the left side (Vector group), those isolated from the right side (NFKBIZ group) showed a reduction in tumor size and weight (Fig. 4A–C). It was then confirmed that the mRNA and protein levels of NFKBIZ in NFKBIZ group were significantly higher than those in the Vector group (Fig. 4D, E). Proliferating Cell Nuclear Antigen (PCNA) is a marker that reflects the state of cell proliferation [19]. We also detected the expression of PCNA in the Vector and NFKBIZ groups. The results revealed that the expression of PCNA in NFKBIZ group was remarkably weaker than that in the Vector group, further confirming the inhibitory effect of NFKBIZ on HCC cells growth (Fig. 4E, F).

To validate the impact of NFKBIZ on HCC metastasis in vivo, an equal number of MHCC97H and Huh7 cells with stable Vector expression and NFKBIZ overexpression were separately injected into the tail vein of nude mice for the construction of lung metastasis model. The in vivo results showed the number of lung metastatic nodules was significantly reduced in NFKBIZ group (Fig. 4G–J). In all, these data confirmed that NFKBIZ effectively suppressed the growth and metastasis of HCC cells in vivo.

### NFKBIZ mediates HCC progression by regulating NFκB signaling transduction

**Fig. 5:**
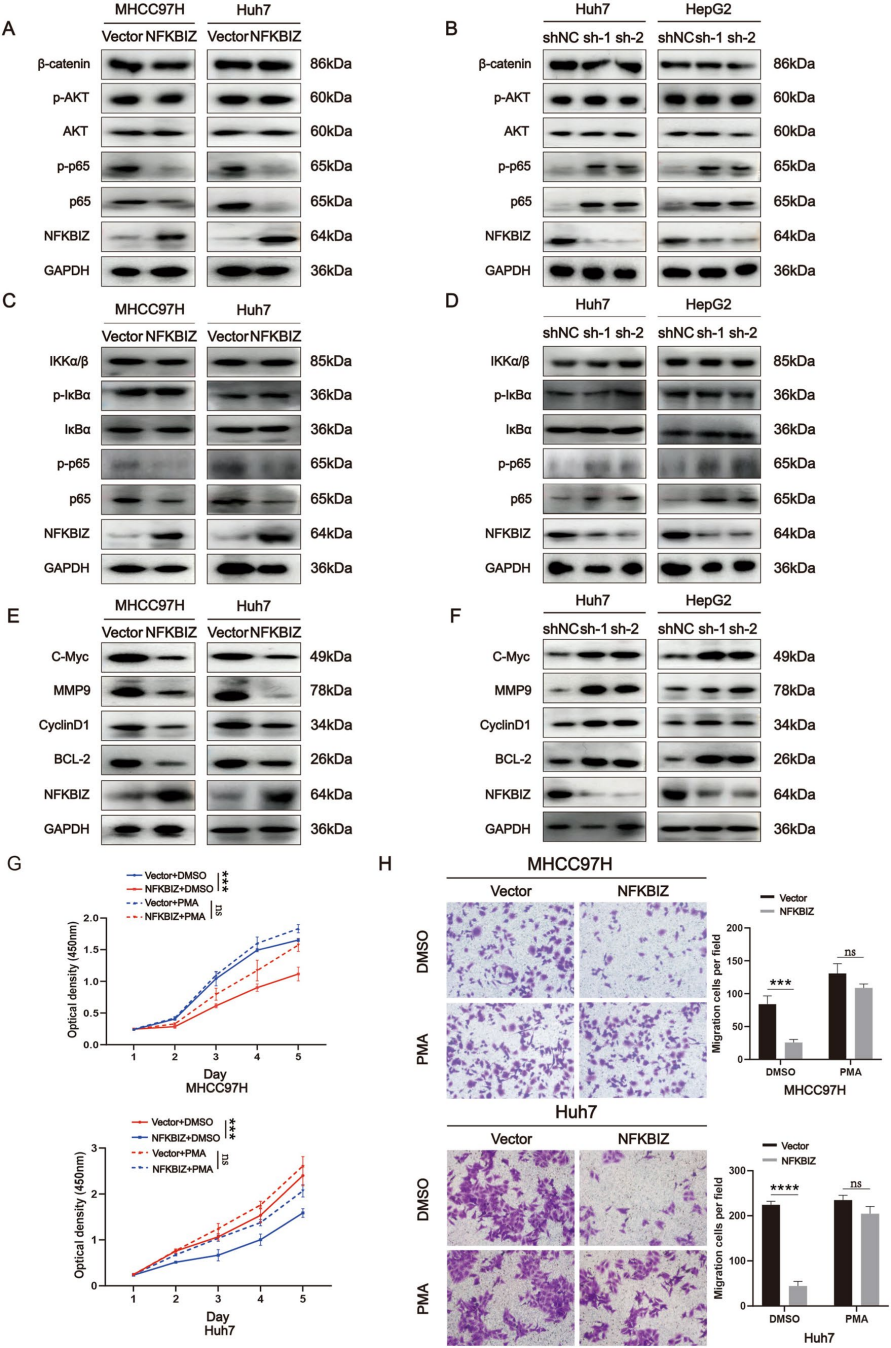
NFKBIZ regulates NFκB signaling transduction to mediate HCC progression. Western blot analysis was performed to determine the protein expressions of β-catenin, AKT/p-AKT, and NFκB in MHCC97H and Huh7 cells with Vector or NFKBIZ overexpression plasmid (**A**), or in Huh7 and HepG2 cells with transient transfections of shNC, sh-NFKBIZ-1, or sh-NFKBIZ-2 plasmid (**B**) for 48 h (*n* = 3 for each group). **C**,**D** The hub target expressions of classical NFκB signaling pathway (IKKα/β, IκBα, p-IκBα, p65 and p-p65) were analyzed via Western blot in HCC cells with the same treatments as A or B. **E**,**F** Western blot was used to determine NFκB targeted genes in HCC cells with the same treatments as **A** or **B**. **G** The proliferation ability was determined by the CCK-8 assay in MHCC97H and Huh7 cells that transfected with Vector or NFKBIZ overexpression plasmid, with or without PMA treatment (50ng/ml) for 24 h (top, Vector + DMSO vs. NFKBIZ + DMSO, *p* = 0.0001, Vector + PMA vs. NFKBIZ + PMA, *p* = ns; bottom, Vector + DMSO vs. NFKBIZ + DMSO, *p* = 0.0010, Vector + PMA vs. NFKBIZ + PMA, *p* = ns, *n* = 3 for each group, two-way ANOVA). **H** Transwell assay showed the migration ability of HCC cells with the same treatment as **G**. Representative images were taken by optical microscope (magnification: 40×, PMA concentration: 50ng/ml). Histograms showing the migration cells per field in these cell lines were also presented (top, Vector + DMSO vs. NFKBIZ + DMSO, *p* = 0.0006, Vector + PMA vs. NFKBIZ + PMA, *p* = ns; bottom, Vector + DMSO vs. NFKBIZ + DMSO, *p* < 0.0001, Vector + PMA vs. NFKBIZ + PMA, *p* = ns, *n* = 3 for each group, two-way ANOVA). * *p* < 0.05, *** *p* < 0.001, **** *p* < 0.0001, ns, no significance, compared to specific control group

To investigate the underlying mechanism of NFKBIZ in regulating HCC progression, we first examined the involvement of Wnt/β-catenin, PI3K/AKT, and NFκB signaling pathways, which were known as important mediators for HCC progression. Western blot results showed that the expression level of p65 and its active form, phosphorylated-p65 (p-p65) were decreased in HCC cells when NFKBIZ was overexpressed. To the contrary, in HCC cells with NFKBIZ transiently silenced, the expression of p65 and p-p65 were increased. However, manipulations on NFKBIZ expression did not affect the expression of β-catenin, AKT, and phosphorylated-AKT (p-AKT) (Fig. 5A, B). Since p65 and p50 formed a dimer to transduce the signal of classical NFκB pathway, we further investigated whether NFKBIZ regulated the expression of the upstream molecules in this pathway. However, the expression of such proteins including IKKα/β, IκBα, p-IκBα, remained unchanged when NFKBIZ was modulated (Fig. 5C, D).

The hyperactivation of the NFκB signaling pathway is suggested to play a crucial role in inflammation, cell apoptosis, and tumor progression [20, 21]. Research has also shown that NFκB is aberrantly overactivated in HCC, but the relevant role and mechanism of this phenomenon are still unclear [22, 23]. C-Myc, MMP9, CyclinD1, and BCL-2 were tumor-related genes that have been identified to be regulated by NFκB signaling [24–27]. To further elucidate the effect of NFKBIZ on NFκB signaling transduction, we examined the protein expression of these genes. As shown in Fig. 5E and F, the expressions of C-Myc, MMP9, CyclinD1 and BCL-2 were decreased in HCC cells with NFKBIZ overexpression, while it turned to the opposite when NFKBIZ was silenced. Previous studies have showed that NFKBIZ inhibited the expression of NFκB downstream genes by binding to NFκB in the nucleus, which was consistent with our results [6]. However, there was no evidence to support the direct regulation of p65 expression by NFKBIZ. Subsequently, we performed an immunofluorescence assay and observed a decrease in the abundance of p65 in both the cytoplasm and nucleus of HCC cells with NFKBIZ overexpression compared to the Vector group (Supplemental Fig. 1**A** and **B**). Finally, we used PMA, an NFκB agonist, to examine whether the effect of NFKBIZ can be reversed. As shown in Fig. 5G and H, the inhibitory effects of NFKBIZ overexpression on HCC cell growth and migration were eliminated upon PMA treatment. Taken together, these results indicate that the elevation of NFKBIZ expression can inhibit NFκB pathways, which may thus ameliorate HCC progression.

### Sorafenib treatment increases the protein degradation of NFKBIZ in HCC cells by enhancing ubiquitination

**Fig. 6:**
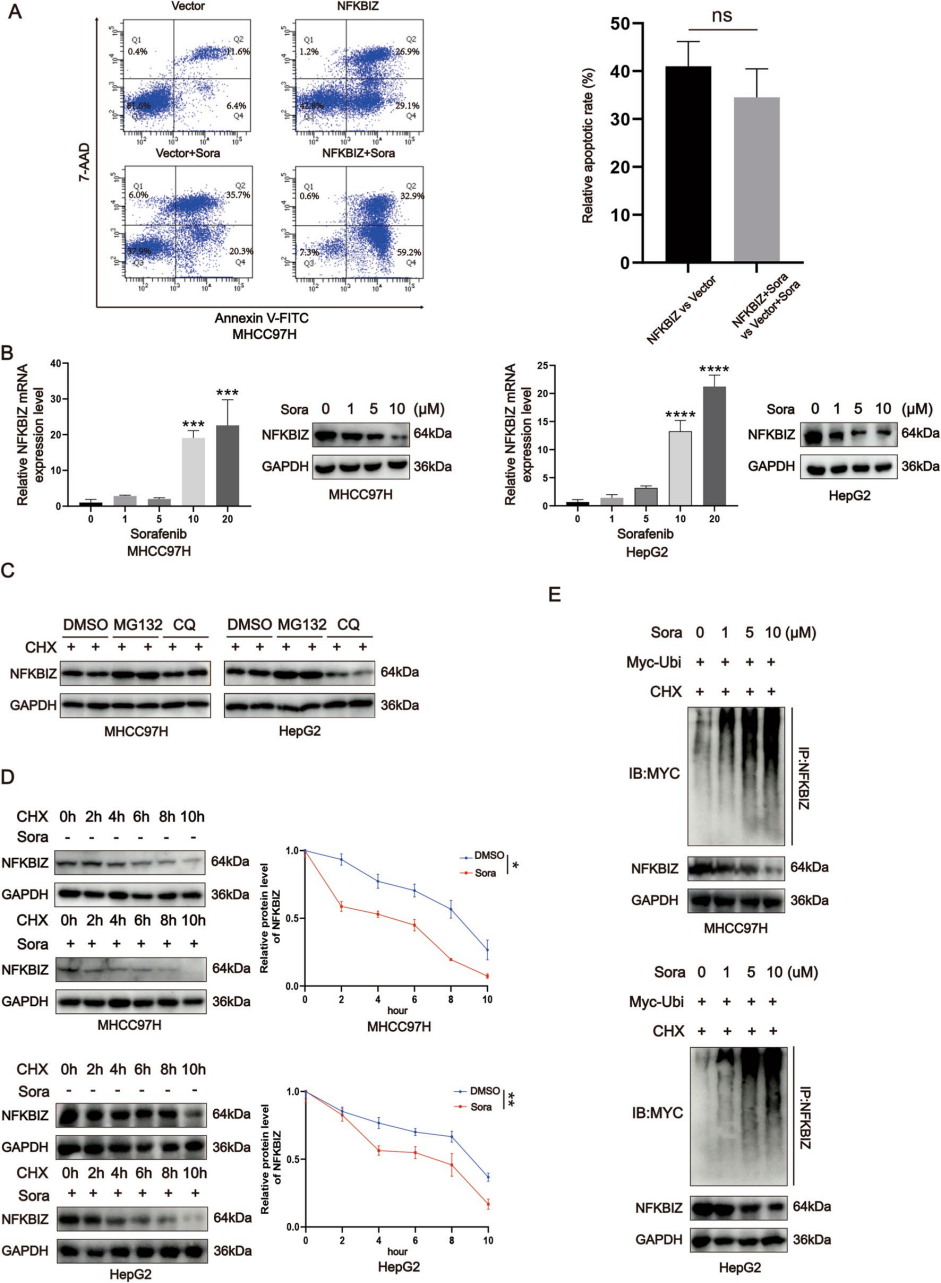
The ubiquitination level of NFKBIZ is enhanced in HCC cells with sorafenib treatment. **A** To examine the effect of NFKBIZ on the apoptosis of HCC cells, flow cytometry was applied in MHCC97H cells transfected with Vector or NFKBIZ overexpression plasmid, accompanied with or without sorafenib treatment for 36 h (left, *n* = 3 for each group). Histogram summarizing the relative apoptosis rate was also presented (*p* = ns, Welch’s *t* test). **B** The mRNA level of NFKBIZ was examined in MHCC97H or HepG2 cells (*n* = 3 for each group, left, *p* = ns, ns, 0.0001, 0.0005, respectively; right, *p* = ns, ns, *p* < 0.0001, *p* < 0.0001, respectively, one-way ANOVA) with different concentrations of sorafenib (0, 1, 5, 10, 20μM) at 36 h, and the protein expression of NFKBIZ in these HCC cells (*n* = 3 for each group) with various sorafenib concentrations (0, 1, 5, 10μM) was examined at 36 h. **C** The expression of NFKBIZ in MHCC97H and HepG2 was showed with DMSO (0.1%), MG132 (50ng/ml) and CQ (50ng/ml) treatment for 24 h (*n* = 3 for each group). **D** MHCC97H and HepG2 was implanted in the 6 well-plate and added with Cycloheximide (CHX) at the time point, 0, 2, 4, 6, 8, 10 h and then the protein of each group (*n* = 3) was extracted. The degradation tendency of NFKBIZ in MHCC97H and HepG2 was presented via Western blot, and the line chart of NFKBIZ degradation was shown (top, *p* = 0.0107; bottom, *p* = 0.0019, one-way ANOVA) (sorafenib concentration: 10μM; CHX concentration: 50ng/ml). **E** The Western blot result of the ubiquitination level of NFKBIZ in these HCC cells with different concentrations of sorafenib added for 24 h (sorafenib concentration: 0, 1, 5, 10μM; CHX concentration: 50ng/ml). * *p* < 0.05, ** *p* < 0.01, *** *p* < 0.001, **** *p* < 0.0001, ns, no significance, compared to specific control group

Sorafenib is a first-line treatment for the advanced HCC. As previously reported, NFκB signaling pathway was abnormally activated in HCC cells with sorafenib resistance [17], where the cell apoptosis cannot be further enhanced. Is it possible that an overexpression of NFKBIZ may inhibit such activation, thus reverse the sorafenib resistance? To explore this, we performed flow cytometry to measure the apoptotic level of HCC cells in NFKBIZ and Vector group with or without sorafenib treatment. Flow cytometry analysis revealed that HCC apoptosis level was significantly elevated when NFKBIZ was overexpressed. Surprisingly, sorafenib treatment accompanied with NFKBIZ overexpression failed to further aggravate HCC cell apoptosis (Fig. 6A). This triggered us to identify whether sorafenib treatment could instead modify the NFKBIZ expression. Indeed, in HCC cells treated with sorafenib, the mRNA level of NFKBIZ was elevated with the increase of sorafenib concentrations. Yet the protein level showed the opposite trend, suggesting it may be regulated by specific post-translational modifications (Fig. 6B).

We then focused on the anti-intuitive decrease of NFKBIZ protein level. As we all know, there are two forms of protein degradation: (1) the ubiquitin–proteasome pathway, and (2) the autophagy-lysosome pathway. To verify the NFKBIZ protein degradation pathway, we detected the expression of NFKBIZ in MHCC97H and HepG2 treated with DMSO, MG132 (an inhibitor of the 26S proteasome), and CQ (an inhibitor of lysosome activity), respectively. A reversal of NFKBIZ protein degradation was observed in HCC cells with the ubiquitin–proteasome pathway inhibited (Fig. 6C). This result was consistent with previous report that NFKBIZ could be degraded by ubiquitination [28]. Further experiment revealed that the degradation of NFKBIZ was increased when sorafenib was added to HCC cells (Fig. 6D). Additionally, we also found an enhancement of NFKBIZ ubiquitination in HCC cells with sorafenib treatment (Fig. 6E). Taken together, we uncovered that HCC cells may develop insensitivity to the anti-tumor drug sorafenib by an increased degradation of NFKBIZ through ubiquitination.

### TRIM16 interacts with NFKBIZ and enhance its ubiquitination to mitigate HCC cell apoptosis

Based on our previous findings, we speculated that the increased ubiquitination level of NFKBIZ may be a result of the upregulation of an E3 ubiquitin ligase in the upstream pathway of NFKBIZ in HCC cells with sorafenib treatment. The tripartite-motif (TRIM) proteins, one of the largest categories of E3s in humans, were of great interest to us due to their role in HCC progression [29]. It has been proposed that TRIMs were strongly associated with the dysregulation of pathways such as JAK/STAT, PI3K/AKT, TGF-β, NFκB, and Wnt/β-catenin in HCC progression [30]. Additionally, aberrant expression of TRIMs is also suggested to be responsible for drug resistance [31].

**Fig. 7:**
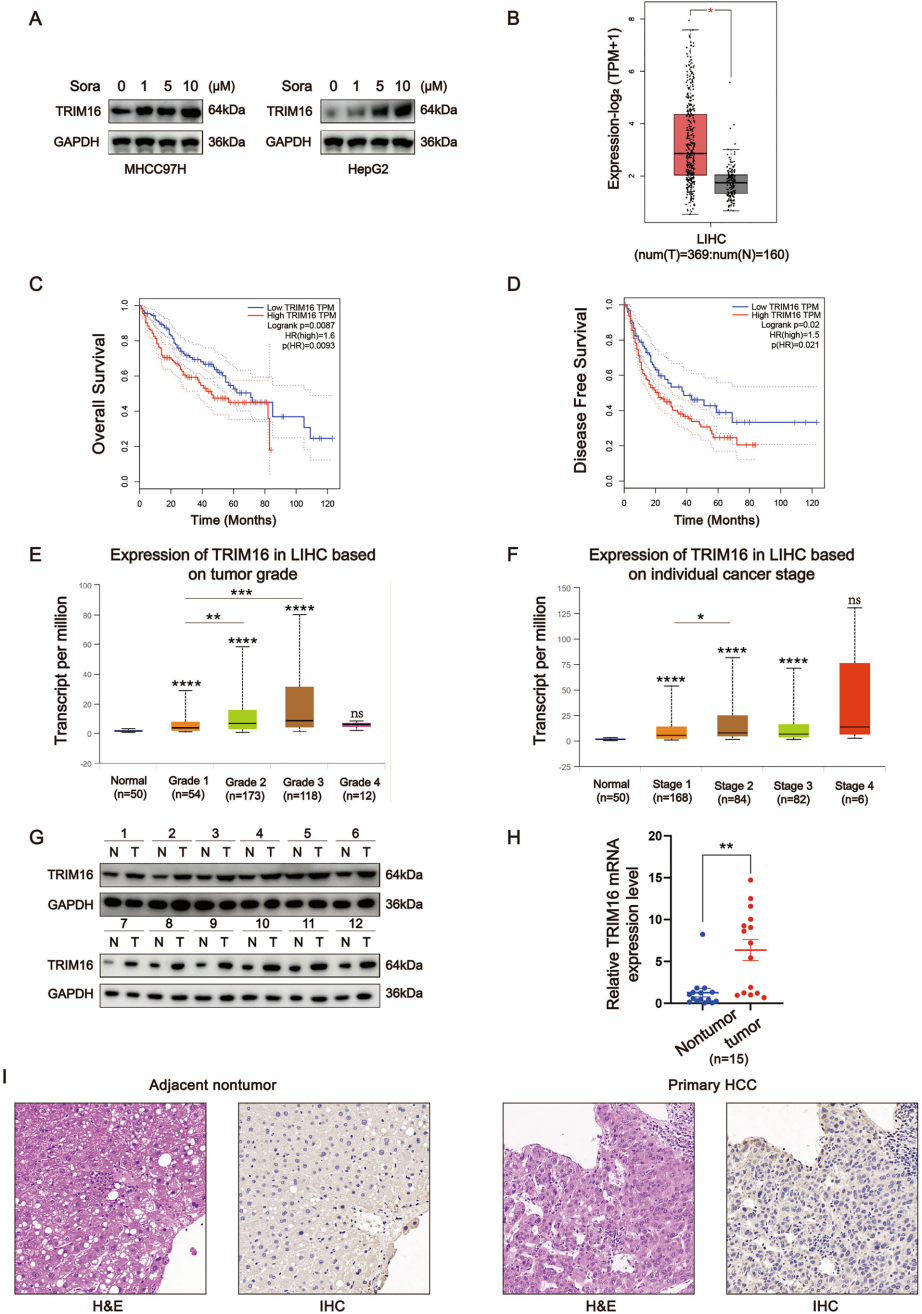
TRIM16 is overexpressed in HCC tissues and indicates a poor prognosis for HCC patients. **A** The expression of TRIM16 in MHCC97H and HepG2 cells with the treatment of different sorafenib concentrations (0, 1, 5, 10μM) was examined. **B** The expression of TRIM16 in LIHC based on sample types in the GEPIA database (T: tumor, *n* = 369; N: normal liver, *n* = 160, *p* < 0.05, Student’s *t* test). **C**,**D** HCC patients were divided into low- and high-TRIM16 group (*n* = 162, respectively) by the cut point, 50%, according to the GEPIA data. Kaplan–Meier survival analysis was used to investigate the effect of TRIM16 on the OS and DFS between the two groups. **E**,**F** The gene expression of NFKBIZ in patients with different HCC grades (*p* (Normal vs. Grade1, Normal vs Grade2, Normal vs Grade3) < 0.0001, *p* (Grade1 vs. Grade2) = 0.0083, *p* (Grade1 vs. Grade3) = 0.0014, *p* (Normal vs. Grade4) = ns, one-way ANOVA) and stages (*p* (Normal vs. Stage1, Normal vs Stage2, Normal vs Stage3) < 0.0001, *p* (Stage1 vs. Stage2) = 0.023, *p* (Normal vs. Stage4) = ns, one-way ANOVA) was presented according to UALCAN database. **G**,**H** Western blot (*n* = 12) and qRT-PCR (*n* = 15, *p* = 0.0010, Mann–Whitney test) were employed to detect the protein and mRNA expression of TRIM16 in adjacent nontumor tissues and HCC tissues from the patients in our cohort. **I** Immunohistochemical assay was used to identify the expression of TRIM16 in adjacent nontumor tissues and HCC tissues. Representative IHC and H&E images were photographed by optical microscope (*n* = 102, magnification: 100×). * *p* < 0.05, ** *p* < 0.01, *** *p* < 0.001, **** *p* < 0.0001, ns, no significance, compared to specific control group

To identify potential upstream targets of NFKBIZ, we screened TRIMs based on their expression levels in HCC tissues through TCGA database and identified TRIM9, TRIM16, TRIM28, TRIM31, TRIM43, and TRIM63 as target genes that are highly expressed in HCC. Among them, only the expression levels of TRIM16 in both MHCC97H and HepG2 were stably elevated with an increase in sorafenib concentrations (Supplemental Fig. 2A and B). Western blot results further verified that the protein levels of TRIM16 appeared to show the same trend as the mRNA levels (Fig. 7A). TRIM16 was found to be upregulated in HCC tissues, and its high expression was associated with poor prognosis, higher tumor grade and stage for HCC patients (Fig. 7B–F). As shown in Fig. 7G–I, compared to adjacent nontumoral tissues, TRIM16 expression showed an obvious increase in HCC tissues of our cohort.

**Fig. 8:**
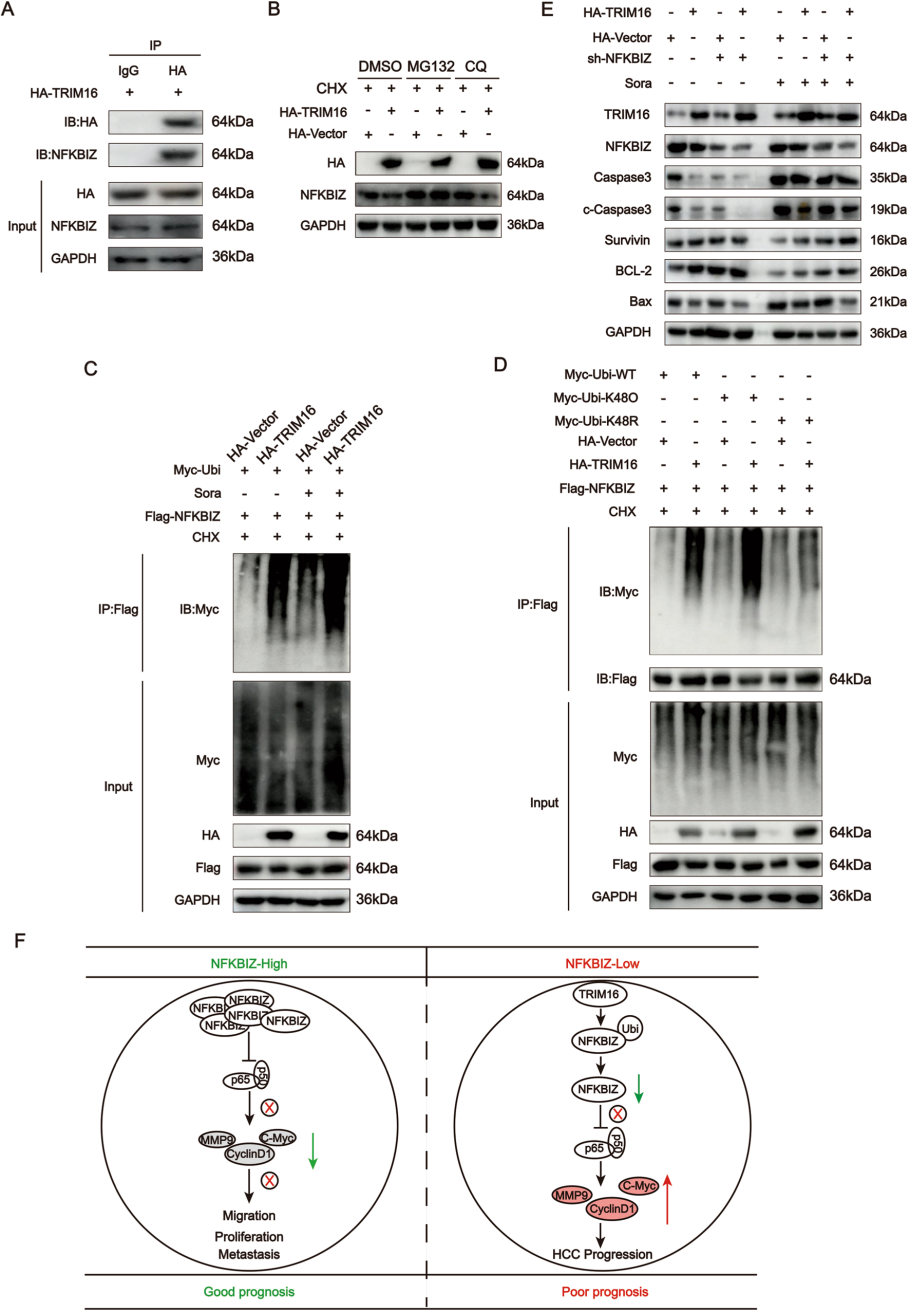
Sorafenib treatment facilitates TRIM16-mediated NFKBIZ ubiquitination. All trails in this figure were examined in Huh7 cells and the sorafenib and CHX concentration used was 10μΜ, 50ng/ml, respectively. **A** The Co-IP result was presented via Western blot, showing the interactions between TRIM16 and NFKBIZ. IB: HA, immunoprecipitation (IP) of HA-TRIM16 by Anti-HA and IgG; IgG is rabbit IgG. Anti-HA (1:1000) was used to detect HA-TRIM16. IB: NFKBIZ, Anti-NFKBIZ was used to detect NFKBIZ; IgG was the negative control. Input represents the whole lysate extracted from Huh7 cells transfected with HA-TRIM16. Anti-HA (1:1000) was used to detect the efficiency of TRIM16 transfection. Anti-NFKBIZ (1:1000) was used to detect the abundance of NFKBIZ. This experiment was repeated three times with similar results. **B** The expression of NFKBIZ in HCC cells with or without TRIM16 overexpression in the condition of DMSO (0.1%), MG132 (50ng/ml) and CQ (50ng/ml) treated for 24 h was examined (*n* = 3 for each group). **C** The ubiquitination level of NFKBIZ in HCC cells with or without TRIM16 overexpression in the condition of sorafenib added or not for 24 h was investigated. **D** The ubiquitination site of NFKBIZ mediated by TRIM16 was examined. **E** The proteins related to cell apoptosis (Caspase3/c-Caspase3, Survivin, BCL-2 and Bax) were examined in HCC cells with or without HA-TRIM16 and sh-NFKBIZ transfection after 48 h (*n* = 3 for each group). **F** The schematic diagram of the study

To verify whether TRIM16 could act as the upstream E3 ubiquitin ligase of NFKBIZ, we conducted Co-IP assays and it demonstrated that TRIM16 interacted with NFKBIZ (Fig. 8A). Subsequently, we discovered that TRIM16 overexpression regulated the ubiquitination degradation of NFKBIZ (Fig. 8B). We further detected effect of TRIM16 on NFKBIZ ubiquitination in HCC cells with or without sorafenib treatment. To our delight, just as expected, TRIM16 directly mediated NFKBIZ ubiquitination, and this regulatory effect was further enhanced under sorafenib treatment (Fig. 8C). The K48 site is known as a protein-ubiquitinated site for further recognition by the 26S proteasome. We found that TRIM16 mediated NFKBIZ ubiquitination at its K48 site rather than other sites (Fig. 8D). In order to elucidate the regulatory role of the TRIM16/NFKBIZ axis in HCC apoptosis, TRIM16 overexpression or/and sh-NFKBIZ plasmid were transfected into HCC cells to detect the expressions of apoptosis-related proteins. As shown in Fig. 8E, in HCC cells transfected with sh-NFKBIZ, the expressions of pro-apoptotic proteins including Caspase3/c-Caspase3 and Bax were decreased, while the expressions of anti-apoptotic markers (Survivin and BCL-2) were enhanced. Such phenomenon also appeared in HCC cells that were transfected with TRIM16 overexpression plasmid, probably due to an increase in NFKBIZ degradation through the direct interaction with TRIM16. Moreover, the expression of TRIM16 was increased in HCC cells with sorafenib treatment, which may thereby reduce the abundance of NFKBIZ, and further hinder the cell apoptosis (Fig. 8E).

Taken together, high NFKBIZ expression tend to inhibit the transcriptional activation of genes regulated by NFκB dimer to mitigate HCC progression, which becomes a significant factor for the better prognosis. Mechanically, TRIM16 could promote NFKBIZ ubiquitination to exacerbate HCC progression, which may be the underlying mechanism of the occurrence of sorafenib insensitivity in HCC clinical treatment (Fig. 8F).

## Discussion

NFKBIZ was initially reported to exhibit similar amino acid sequences with BCL-3 and other members of the IκB family [6]. Owing to its induction by Interleukin-1, research primarily centered on the involvement of NFKBIZ in inflammation and immune responses. Studies on diseases with autoimmune and inflammatory disorder such as psoriasis, Crohn’s disease, and ulcerative colitis shed light on the pivotal effects NFKBIZ exerted. Additionally, it has garnered significant attention over past years that NFKBIZ plays an important role in the tumorigenesis of many diseases, such bladder cancer, rectal cancer and DLBCL. However, there was a scarcity of research investigating NFKBIZ function in HCC. Our results consistently confirmed the reduced expression of NFKBIZ in HCC compared to adjacent nontumoral tissues, aligning with TCGA database findings. Notably, our results from IHC showed that NFKBIZ expressed in both cytoplasm and nuclear, which differed from the results of previous research that NFKBIZ only accumulated in nuclear [6]. We speculated that, during the transformation of liver cells to HCC cells, liver cells generally located in a chronic inflammation context leading to high NFκB abundance and overactivation of NFκB signaling pathway. Hence, upregulating NFKBIZ expression may be a way for liver cells to negatively modulate NFκB activity and subsequently mitigate consecutive inflammatory response. Remarkably, our follow-up data revealed that HCC patients with high NFKBIZ expression exhibited lower infection rate of HBV, and this may be a clue to support our speculation. In fact, HBV infection has long been considered as crucial risk factor for HCC development [32]. For instance, liver fibrosis induced by chronic HBV infection greatly affects the recurrence in HCC patients [33]. The findings of other studies also showed that patients with HBV-related HCC had larger maximum tumor sizes [34] and HCC patients with high serum HBV DNA load were more prone to have a recurrence [34], which were consistent with our research. However, relative research about NFKBIZ and HBV infection was limited, which remained to be further explored.

Subsequently, the effects of NFKBIZ on the proliferative, invasive, and migratory abilities of HCC cells were explored. Our experimental data revealed that NFKBIZ overexpression significantly inhibited such biological functions of HCC cells, while NFKBIZ silencing generated the opposite effect. Additionally, we focused on the EMT, a process intricately associated with tumor metastasis, and demonstrated that NFKBIZ could inhibit this process to impede HCC progression.

To explore the underlying mechanism through which NFKBIZ regulates HCC cells, we focused on three pathways known to be abnormally activated during HCC development: PI3K/AKT, Wnt/β-catenin, and NFκB. The results revealed that NFKBIZ negatively regulated p65, a hub target of the classic NFκB signaling pathway. Consequently, downstream targets of NFκB that promoted tumor progression were downregulated due to NFKBIZ overexpression. Previous studies suggested NFKBIZ bound to p65/p50 dimers in the nucleus, thereby inhibiting downstream gene transcription of NFκB. Our results further indicated that NFKBIZ regulated the expression of p65. To delve deeper into the mechanisms involved, we conducted immunofluorescence trials to investigate p65 expression in MHCC97H and Huh7 cells stably overexpressing NFKBIZ. The results showed that compared to the control group, the abundance of p65 in both the cytoplasm and nucleus in NFKBIZ overexpression group was downregulated, which also confirmed our findings of Western blot. However, this observation may also be attributed to an indirect regulation by the downstream genes of NFKBIZ. Of note, studies have reported that NFKBIZ could directly interact with p50 and p52, while there was still no established research to support the regulation of p65 by NFKBIZ [8]. Our findings may provide new insight into the regulation of NFκB. Notably, some reports have proposed the activation of NFκB pathway alleviates HCC progression by mitigating inflammatory stress, implying the complex network between NFκB signaling and HCC carcinogensis [35, 36]. As previously reported, unlike other IκB family members, NFKBIZ could not mediate the transduction of NFκB into the nucleus. Intriguingly, our immunofluorescence results suggested that the abundance of p65 in the nucleus of HCC cells fluctuated more significantly than in the cytoplasm, warranting further investigation to unravel the implication of such phenomenon in HCC.

Given the prevalent overactivation of the NFκB pathway in HCC and its link to cellular anti-apoptosis mechanisms provoked by inflammation, we explored the impact of NFKBIZ on HCC apoptosis. For this purpose, we introduced sorafenib, a first-line drug for advanced HCC treatment, which is known to induce HCC apoptosis but with increasing drug resistance challenges correlated with an abnormal activation of NFκB. We revealed the inconsistency between NFKBIZ mRNA and protein expression under sorafenib treatment. We speculated NFKBIZ upregulation may work in synergy with sorafenib to enhance HCC apoptosis, making NFKBIZ a potential target to sensitize HCC to sorafenib. However, to counteract apoptosis induction, HCC cells may promote NFKBIZ degradation to resist sorafenib and result in the activation of anti-apoptotic targets of NFκB. This cascade reaction may further activate the MAPK pathway through a bypass mechanism, exacerbating the anti-apoptotic abilities of HCC cells. We also identified TRIM16 as the specific E3 of NFKBIZ to promote its degradation. Noteworthily, the expression of TRIM16 in HCC cells enhanced with increasing sorafenib concentrations. This observation could be the reason for the reduced protein abundance of NFKBIZ with sorafenib added in HCC and provided new insights into the mechanism of sorafenib insensitivity.

However, there are still limitations in our present study. For instance, despite we have unveiled the effects and mechanisms of NFKBIZ on regulating HCC cells, less evidence was provided for that of NFKBIZ downregulation, especially in vivo. Considering the limitations of our xenograft model, it’s theoretically and technically impossible to isolate any relative normal tissue as a negative control. Therefore, we could only try to reveal the direct effect of decreased NFKBIZ expression on HCC malignant behaviors in vivo, by comparing the tumor tissue from both sides of our xenograft mice with HepG2 or MHCC97H cells stably transfected with sh-NFKBIZ on the right dorsal side and those cells with sh-NC on the left side (Supplemental Fig. 3). This turned out to be statistically insignificant, and we suspect it might be the rather complicated environment in vivo that has concealed the real effects of our knock-down operations. It’s also a bit disappointing that we did not find any significant correlation between NFKBIZ expression level and extrahepatic metastasis in our cohort, although NFKBIZ downregulation in native HCC cells may promote tumor metastasis. Perhaps it won’t be the case in other clinical cohorts, or due to the complexity of the human bodies that could activate other compensatory mechanisms. In either case, multicenter trails are warranted to validate the impacts of NFKBIZ on tumor metastasis in the real world.

In conclusion, our study revealed that NFKBIZ mediated HCC proliferation, invasion and metastasis by regulating the NFκB signaling transduction. TRIM16 directly interacted with NFKBIZ and promoted the ubiquitination, which mitigates the apoptosis-inducing effect of sorafenib on HCC cells. Our findings underscored the TRIM16/NFKBIZ/p65 axis played a pivotal role in regulating HCC progression and NFKBIZ as a promising therapeutic and prognostic target for HCC, as well as a potential target to enhance sorafenib sensitivity for HCC treatment.

### Supplementary Information

Below is the link to the electronic supplementary material.

Supplementary file1 (JPG 912 kb)

Supplementary file2 (JPG 1915 kb)

Supplementary file3 (PDF 125745 kb)

## Data Availability

The data that support the findings of this study are available from the corresponding author upon reasonable request.
