# Supplementary figures and images for: NFKBIZ regulates NFκB signaling pathway to mediate tumorigenesis and metastasis of hepatocellular carcinoma by direct interaction with TRIM16
Source: Cell Mol Life Sci. 2024 Apr 6;81(1):167. doi: 10.1007/s00018-024-05182-7 (PMC10998794; doi:10.1007/s00018-024-05182-7)

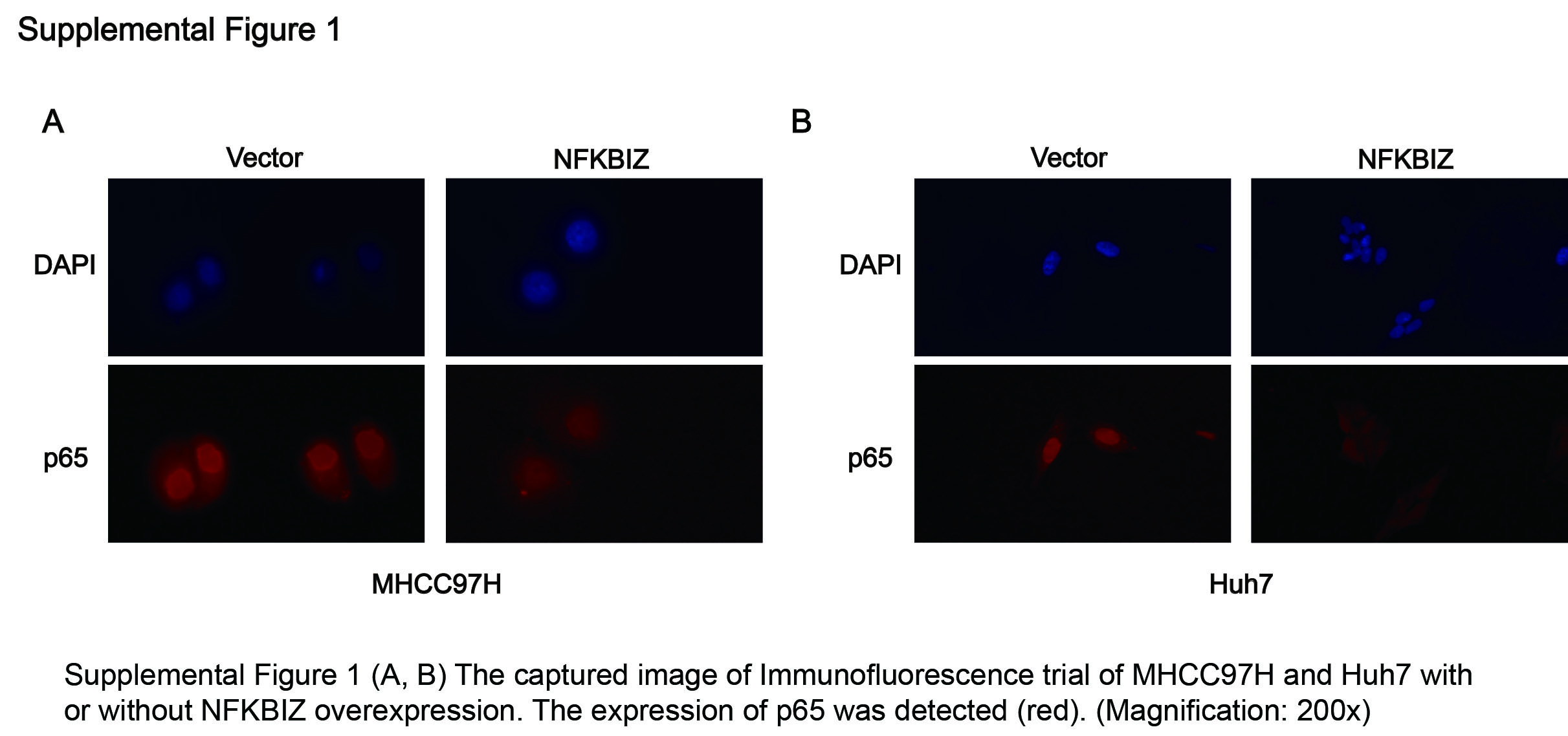

Supplement: Supplementary file 1 — Supplementary file1 (JPG 912 kb) [file 18_2024_5182_MOESM1_ESM.jpg]

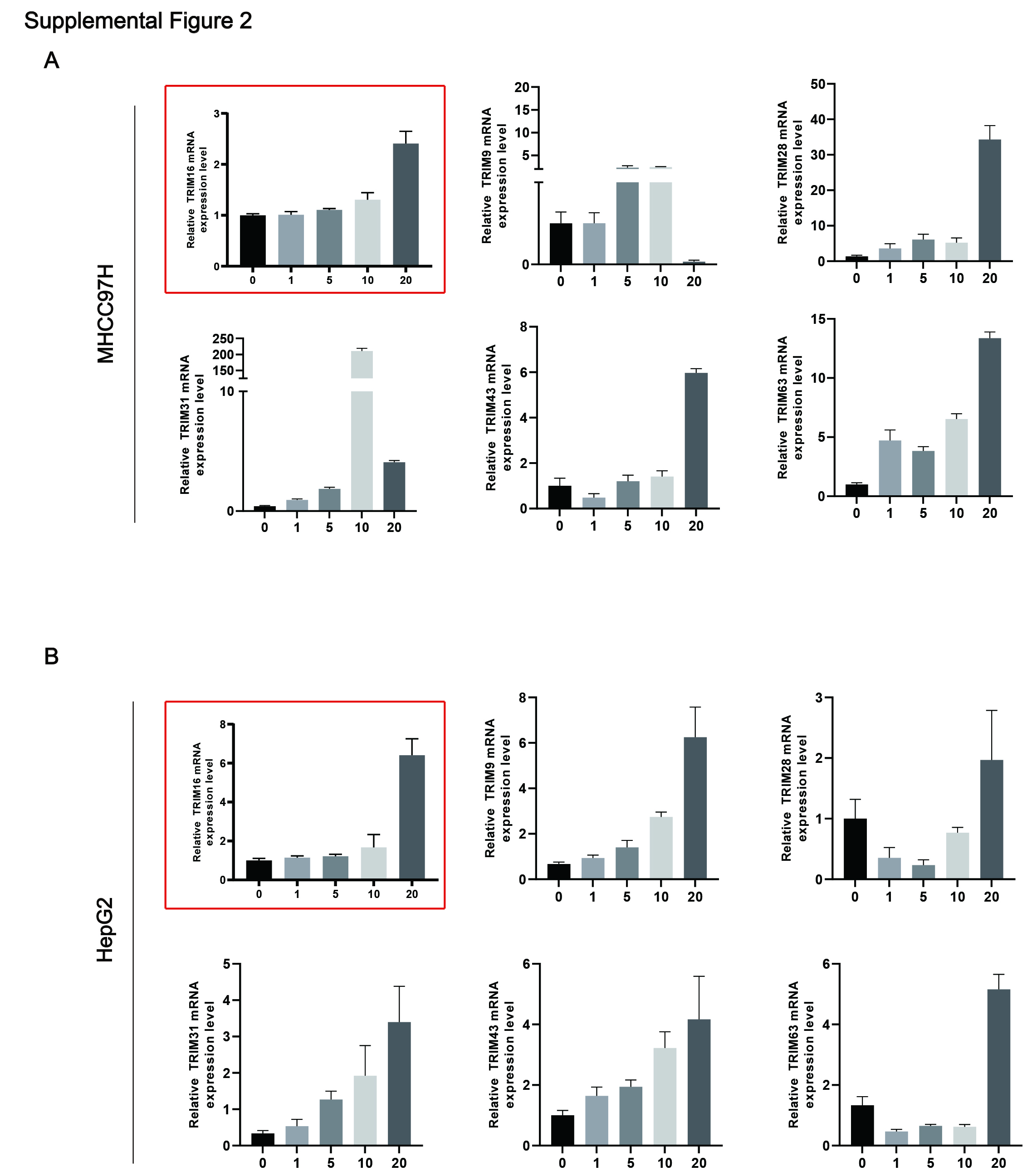

Supplement: Supplementary file 2 — Supplementary file2 (JPG 1915 kb) [file 18_2024_5182_MOESM2_ESM.jpg]
