# Supplementary material for: NFKBIZ regulates NFκB signaling pathway to mediate tumorigenesis and metastasis of hepatocellular carcinoma by direct interaction with TRIM16
Source: Cell Mol Life Sci. 2024 Apr 6;81(1):167. doi: 10.1007/s00018-024-05182-7 (PMC10998794; doi:10.1007/s00018-024-05182-7)
Supplement: Supplementary file 3 — Supplementary file3 (PDF 125745 kb) [file 18_2024_5182_MOESM3_ESM.pdf]

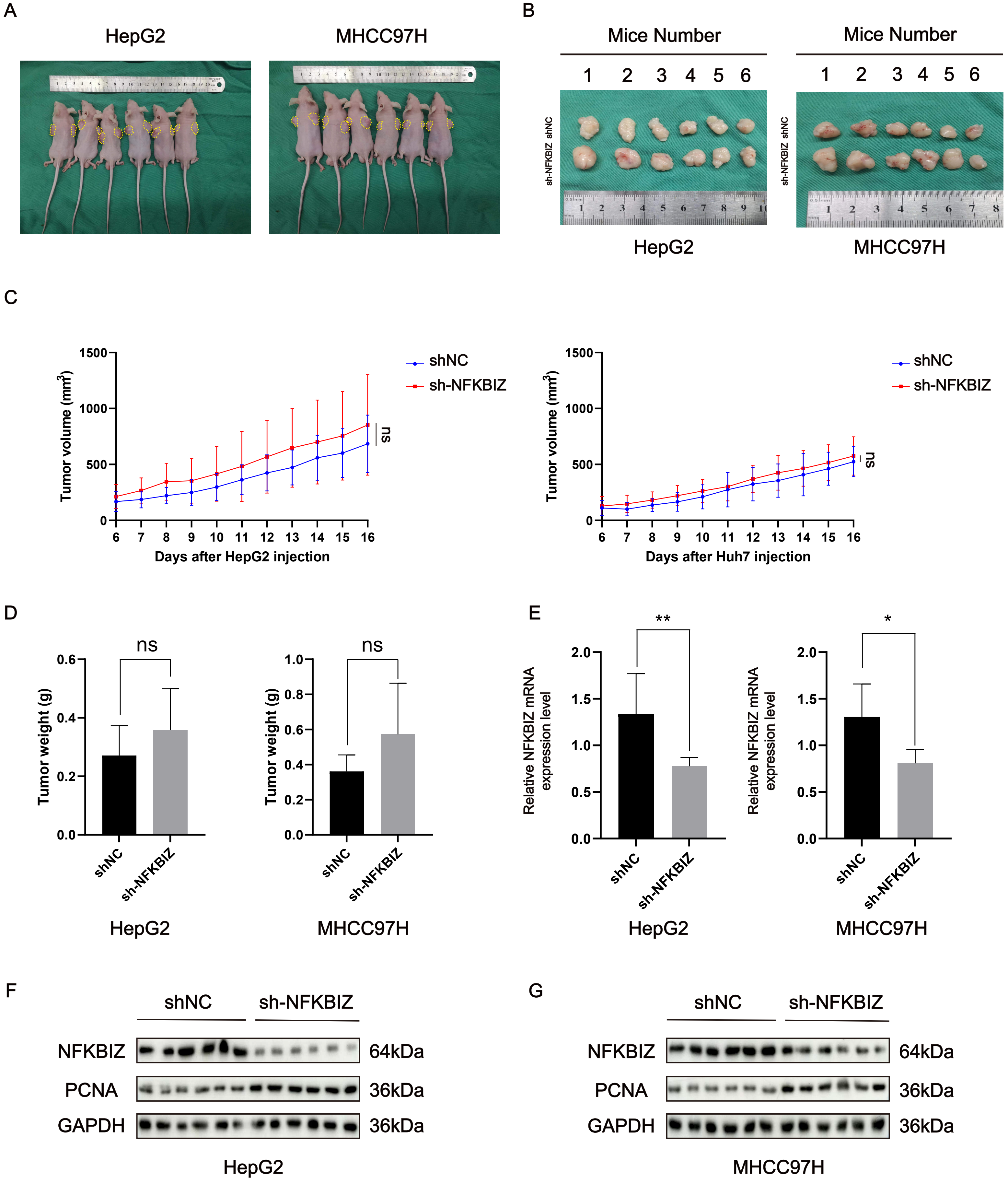

(A,B) The images of BALB/c-nude mice in each group (n=6) and the tumor nodules at scheduled time point after subcutaneous injections of HepG2 and MHCC97H with shNC (left) or sh-NFKBIZ (sh-1) stably transfected (right). (C) The line chart of the tumor volume of each group.(D) The mRNA level of NFKBIZ in tumor tissues extracted from subcutaneous mouse xenograft models in each group . (E) The weight of tumors in each group. (F, G) NFKBIZ and PCNA expressions of tumor from subcutaneous mouse xenograft models in each group.
